# Pseudonymization for research data collection: is the juice worth the squeeze?

**DOI:** 10.1186/s12911-019-0905-x

**Published:** 2019-09-04

**Authors:** Florian Kohlmayer, Ronald Lautenschläger, Fabian Prasser

**Affiliations:** Institute of Medical Informatics, Statistics and Epidemiology, University Hospital rechts der Isar, Technical University of Munich, Munich, Germany

## Abstract

**Background:**

The collection of data and biospecimens which characterize patients and probands in-depth is a core element of modern biomedical research. Relevant data must be considered highly sensitive and it needs to be protected from unauthorized use and re-identification. In this context, laws, regulations, guidelines and best-practices often recommend or mandate pseudonymization*,* which means that directly identifying data of subjects (e.g. names and addresses) is stored separately from data which is primarily needed for scientific analyses.

**Discussion:**

When (authorized) re-identification of subjects is not an exceptional but a common procedure, e.g. due to longitudinal data collection, implementing pseudonymization can significantly increase the complexity of software solutions. For example, data stored in distributed databases, need to be dynamically combined with each other, which requires additional interfaces for communicating between the various subsystems. This increased complexity may lead to new attack vectors for intruders. Obviously, this is in contrast to the objective of improving data protection. What is lacking is a standardized process of evaluating and reporting risks, threats and countermeasures, which can be used to test whether integrating pseudonymization methods into data collection systems actually improves upon the degree of protection provided by system designs that simply follow common IT security best practices and implement fine-grained role-based access control models. To demonstrate that the methods used to describe systems employing pseudonymized data management are currently heterogeneous and ad-hoc, we examined the extent to which twelve recent studies address each of the six basic security properties defined by the International Organization for Standardization (ISO) standard 27,000. We show inconsistencies across the studies, with most of them failing to mention one or more security properties.

**Conclusion:**

We discuss the degree of privacy protection provided by implementing pseudonymization into research data collection processes. We conclude that (1) more research is needed on the interplay of pseudonymity, information security and data protection, (2) problem-specific guidelines for evaluating and reporting risks, threats and countermeasures should be developed and that (3) future work on pseudonymized research data collection should include the results of such structured and integrated analyses.

**Electronic supplementary material:**

The online version of this article (10.1186/s12911-019-0905-x) contains supplementary material, which is available to authorized users.

## Background

The collection of fine-grained personal health data has become an important element of biomedical research, which is required to obtain characterizations of patients and probands in necessary breadth and depth. While data is collected at increasing rates, privacy concerns are increasing as well [1]. However, the number of health data breaches is growing [2] and there is significant public pressure to ensure that the privacy of patients and probands is protected [3]. On the regulatory level, the protection of research data has also been addressed, e.g. in the General Data Protection Regulation of the European Union [4, 5], the European Recommendation on Research on Biological Materials of Human Origin [6], and the Privacy Rule of the US Health Insurance Portability and Accountability Act (HIPAA) [7]. Adequate privacy protection becomes even more challenging when annotated biosamples need to be managed in addition to research data [6, 8].

As a primary data protection mechanism, laws, regulations, guidelines and best-practices often recommend or mandate *pseudonymization.* This means that directly identifying data of patients and probands (e.g. names and addresses) is stored separately from data which is primarily needed for scientific analyses [9–12]. The ultimate goal of this process is to ensure that sensitive research data cannot be attributed to a natural person without combining it with its associated identifying information. This implies that re-identification can be prevented by making sure that attackers cannot gain integrated access to both research data and associated directly identifying data. The data stored in the various databases is typically linked to each other using random alphanumerical identifiers (pseudonyms) [13] but further approaches, e.g. using cryptographic schemes, have also been proposed.

**Fig. 1:**
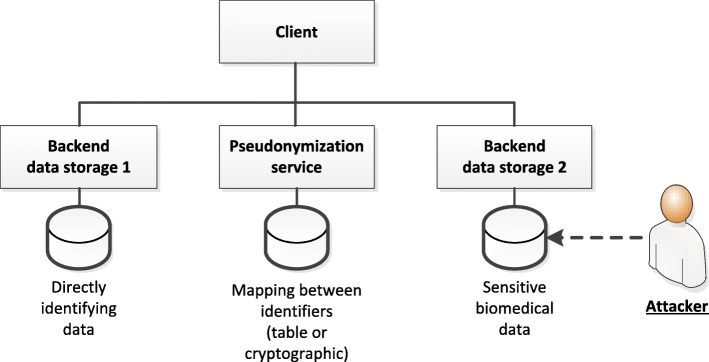
Basic attack scenario addressed by pseudonymization

A schematic overview of the basic attack scenario addressed by research data pseudonymization is shown in Fig. 1. As can be seen, it is assumed that the adversary explicitly attacks either the database storing identifying data or the database storing research data and that research data cannot be identified while identifying data is not sensitive. Some concepts even introduce additional services that perform further pseudonymization steps (e.g. mapping first-tier pseudonyms to second-tier pseudonyms) and implement hardware-level protection for this service using Smart Cards [14, 15]. We emphasize that the figure illustrates a common perspective, which has found its way into many solutions, national legislations, e.g., in Germany [9] Italy [10] and in the United Kingdom (UK) [11, 16], and into data protection guidelines and best practices [12].

## A critical appraisal of Pseudonymization for research data collection

Although the basic understanding of pseudonymization outlined in the previous section can be positively appraised for clearly modeling and mitigating a few specific types of anticipated attacks, it is also obvious that the technique falls very short in terms of protecting data against a broad spectrum of realistic threats. In recent years it has been shown that clinical data, such as diagnoses or laboratory values, also increase the degree of distinguishability of individuals significantly, which can be used for re-identification [17]. This is particularly true for high-dimensional and sparse data collections, which are common in biomedical research [18]. This has led to a change in the perception of the degree of protection provided by pseudonymization methods, which is also reflected in new legislation. For example the European General Data Protection Regulation considers data pseudonymous if it “can no longer be attributed to a specific data subject without the use of additional information” [5].

Pseudonymization has been implemented into solutions for the secondary use of data (cf. Vanderbilt’s Synthetic Derivative [19]) and when research data is collected for project-specific purposes [20]. However, in the latter case, implementing pseudonymization can significantly increase the attack-surface of a system. The main reason is that, in data collection systems, authorized re-identification is not an exceptional but a common procedure. This is particularly true in longitudinal data collection where person-identifying data, such as names and insurance numbers, is required to verify the identity of patients or probands prior to data entry. In this case, the physicians or researchers are often directly involved in the process, which implies a lower potential for automating and streamlining processes. At the same time, systems for collecting research data and biospecimens that are implemented based on separated data pools can become significantly more complex. Linking distributed data items with each other is often difficult, especially when additional services for managing pseudonyms (two-tier pseudonymity) are used [20–22]. Some guidelines even require that data is only combined on the client-side [12]. This increases complexity in terms of the number of interfaces that need to be implemented for communicating between the various subsystems [20, 23]. Often, it is also required that the different subsystems are not only separated on the technical but also on the organizational level [12]. This makes it difficult to maintain and keep all components up-to-date [20, 24]. Consequently, new attack vectors are potentially opened up and the overall attack surface for intruders may be increased. This is obviously in stark contrast to the initial aim of improving data protection by implementing pseudonymization. In IT-security this problem is typically summarized in the wise saying that “complexity is an enemy of security” [25].

Finally, the different data modules that are to be separated from each other often correspond surprisingly well with the responsibilities of different personnel involved in research data collection (e.g. identity management, data entry and biosample management [12]). This suggests that pseudonymization is being used to implement the need-to-know principle, i.e. to control which types of data are accessible to which groups of users in which context. However, this problem can be solved with much less complexity and without distributing data by implementing fine-grained role-based access models. Also, pseudonymization concepts do not adequately distinguish between threats from internal and threats from external attackers [12]. We argue that structured descriptions of systems, their underlying assumptions and standardized processes for evaluating and reporting risks, threats and countermeasures are lacking. Future work should aim at clearly showing that integrating pseudonymization methods into data collection systems actually improves data protection compared to collecting data using a single, monolithic and properly hardened system, with a model of rights and roles that adequately reflects the need-to-know principle [26].

## Literature review

### Method

To get an impression of how the aforementioned aspects are addressed in the literature and when building systems for research data capture, we conducted a review of recent articles describing systems for collecting research data using pseudonymized data storage. We note that our selection is not a representative sample of papers about studies employing pseudonymization, but a selection of papers presenting concrete systems while emphasizing security and privacy aspects. For a description of the exact search and selection process, we refer the interested reader to Additional file 1.

We analyzed whether the authors have conducted a structured risk and threat analysis and to which extent they address basic information security methods in this process. There are various methodologies, guidelines and standards for securing information systems, also in the biomedical domain [27]. In general, information security aims at minimizing the impact of attacks and it comprises the management of appropriate security measures that protect against various threats. To achieve this goal, the well-known standard ISO 27000 has formulated six basic security principles [28], an overview of which is shown in Table 1.

**Table 1:** Overview of basic security properties defined by ISO 27000 (Descriptions from [28])

Property	Description
1. Authenticity	“Property that an entity is what it claims to be”
2. Integrity	“Property of protecting the accuracy and completeness of assets”
3. Accountability	“Responsibility of an entity for its actions and decisions”
4. Confidentiality	“Property that information is not made available or disclosed to unauthorized individuals, entities, or processes”
5. Availability	“Property of being accessible and usable upon demand by an authorized entity”
6. Authorization	“Approval that is granted to a system entity to access a system resource”

The aim of pseudonymization is to make the identity of data subjects confidential to unauthorized actors. Hence, confidentiality, which means that no information is disclosed to entities which are not supposed to have access to it, is an important security principle in our context. However, to determine whether an entity is supposed to have access, authenticity of the entity has to be ensured, which implies that measures have to be implemented that prevent the spoofing of identities. Even if such methods have been put in place, it has to be determined which resources may be accessed by which entity. To this end, an authorization concept is needed in which rights and roles have been designed carefully (based on the need-to-know principle) and measures must be implemented to prevent the unauthorized elevation of privileges. To implement all these mechanisms in a reliable manner, the integrity of a system and its data must be protected by methods that prevent adversaries from tampering. To introduce a barrier against insider-attacks and tampering in general also legitimate users must be held accountable for their actions, e.g. by monitoring the system and keeping a log of interactions and changes. Finally, system availability is important because a Denial-of-Service attack can, for example, be used to hide other attacks from users and system administrators.

### Results

Our selection resulted in 12 articles. The earliest approach dates back to 2000, but we also found more current papers from which the most recent ones were published in 2015. The selection solely comprised articles from Europe and 75% of the articles have been written by first authors working at German institutions. This is likely a consequence of data pseudonymization being required by many European data protection laws and official recommendations, e.g. in Germany [9], Italy [10] and in the UK [11, 16]. The high number of German publications is likely related to particular public concerns about data privacy in Germany [29]. Also, many of the German articles, i.e. [20, 22, 30], are based on the generic data protection scheme developed by the German association TMF, Technology, Methods and Infrastructure for Networked Medical Research [12], which is well-known throughout Germany and which has led to the broad adoption of data pseudonymization principles.

The systems described in the articles focus on a wide variety of different research areas and applications. Gulcher et al. presented a system for collecting research data and biospecimens for disease-based gene discovery projects that is supervised by the Data Protection Commission of Iceland [21] (see also [31]). Pommerening et al. described an infrastructure which enables longitudinal studies involving medical data, genetic data and data for managing collections of biomaterials [32]. Eggert et al. presented an approach for collecting data and biomaterial for a research project on Parkinson’s disease [33]. Angelow et al. described a solution for central biosample and data management in a project investigating inflammatory cardiomyopathy [34]. The approach presented by Spitzer et al. utilizes pseudonymization to secure a web-based teleradiology platform for exchanging digital images between authorized users [23]. Dangl et al. have implemented a solution for pseudonymization in the context of an IT-infrastructure for biospecimen collection and management in an academic medical center [22]. Neubauer et al. presented a solution, in which smart cards allow patients to control the re-identification process [14]. Benzschawel and Da Silveira developed a multi-level privacy protection scheme for a national eHealth platform [35]. Demiroglu et al. described a system for a large-scale research project in the area of psychiatric genetics [36]. Majchrzak and Schmitt described a web-based documentation system for long term observations of patients with nephronophtisis [24]. Aamot et al. presented a system which implements sample and data management in translational research for oncology patients [30]. Finally, we have presented a generic solution for pseudonymized data and biosample collection which has been used to implement two research registries [20].

The most frequently described measures that addressed **authenticity** were password protection and server certificates. Almost all articles mentioned both methods, while server certificates were typically used as part of implementing Transport Layer Security (TLS), a well-known cryptographic protocol for secure data exchange on the Internet. The description from [23] did not mention the use of password protection and [33] did not address server certificates. Neither [22] nor [21] addressed any of both measures. Further methods that were described in the reviewed articles included two-factor authentication and the use of password policies. The protection of system **integrity** was often addressed by using an audit trail and logging facilities as well as integrity-protected data transmission. The latter was, again, typically a result of implementing TLS for data exchange. The former was described in [20, 22, 24, 33, 34], while the latter was addressed by all articles with the exception of [21, 22, 33]. Further methods mentioned in the articles included user input validation and sandboxing of system components. The most frequent measures which addressed **accountability** were the use of audit trails and logging facilities as well as organizational and legal processes. The latter included using data use agreements to hold users liable for their actions and implementing data access committees or ethics committees, which control access to data and the setup of research projects. Such measures were described in all articles, with the exception of [14, 23, 35, 36]. **Confidentiality** is the most important security principle in our context. The two most commonly described measures were encrypted data at rest and encrypted data at transit. The latter was, again, typically a result of using TLS, which was described by all articles except [21, 22, 33]. Encrypting data at rest, e.g. in databases, was addressed by all articles with the exception of [22, 32, 36]. The protection of system **availability** was most frequently addressed with the following two measures: backups and firewall. However, many articles mentioned neither of both methods, in particular [21–24, 30, 32, 35]. One article, [14], did not address firewalls, while [33, 36] did not describe backups. In some articles, load balancing was mentioned. Finally, **authorization** was most often addressed by organizational and legal processes and by implementing role-based access control. The latter was described in [14, 20, 22, 23, 34–36]. Only three articles mentioned all security principles: [20, 33, 34].

We emphasize that our analysis only focused on the measures mentioned in the articles, which are not necessarily identical with the measures that have been implemented. As such, the results of our analysis do not describe the degree of protection provided by the individual systems and they cannot be used as a basis for such comparisons. The results show, however, that there is a significant heterogeneity within system descriptions pointing towards a lack of a common methodology. An overview of the results is presented in Table 2.

**Table 2:** Overview of security properties explicitly addressed by mentioning protection mechanisms in the articles considered

	Property							
Ref	Year	Country	Authenticity	Integrity	Accountability	Confidentiality	Availability	Authorization
[14]	2011	Austria	x	x		x	x	x
[20]	2015	Germany	x	x	x	x	x	x
[21]	2000	Iceland			x	x		
[22]	2010	Germany		x	x			x
[24]	2012	Germany	x	x	x	x		
[30]	2013	Germany	x	x	x	x		
[32]	2006	Germany	x	x	x	x		
[33]	2007	Germany	x	x	x	x	x	x
[34]	2008	Germany	x	x	x	x	x	x
[23]	2009	Germany	x	x		x		x
[35]	2011	Luxemb.	x	x		x		x
[36]	2011	Germany	x	x		x	x	x

Finally, we analyzed whether the authors have presented structured analyses of threats and countermeasures taken. We found that only three articles contain the results of such analyses: [14, 20, 30], which have all been published in recent years. However, typically they did not provide details in sufficient depth and no article has presented a structured analysis of risks derived from threats and presented evidence that the system architecture and measures implemented are really adequate for achieving their objective.

## Summary and recommendations

In this work, we critically appraised the implementation of pseudonymization into research data collection processes. We argued that a comprehensive methodology for evaluating and reporting risks, threats and countermeasures in this context is lacking. To demonstrate this, we analyzed recent literature on the topic and found that descriptions are heterogeneous and ad-hoc. The results are consistent with observations by Neubauer et al. that current pseudonymization architectures are based upon an implicit threat model which has not yet been formalized [14] and by Deng et al. that it needs to be clarified which entities are to be protected from which threats [37].

Some articles referenced and implemented the TMF concept [20, 22, 30] and one article [20] referenced the standard ISO/TS 25237, which is a technical specification on data pseudonymization [13]. Both guidelines cover different scenarios in which pseudonymized data must be re-identified, but they do not relate the frequency of such events, alternative implementations or the degree of automatization to the degree of protection provided. Moreover, it is argued frequently that pseudonymization protects data from insider attacks [38]. However, such attacks can also be mitigated by implementing a sound authorization concept. With such a mechanism in place, data can be stored in a single database which can be sealed and protected from external threats in a robust and reliable manner by following well-known best-practices. This has the potential to be much more secure, compared to implementing and protecting a complex distributed system which needs to provide various interfaces to implement complex pseudonymization schemes consisting of multiple databases.

As a first step towards improving the situation, we recommend that articles describing systems for pseudonymized data management present structured analyses of threats and countermeasures taken against internal and external attackers with different motives as well as capabilities and consider users with different degrees of trust. In future work, the STRIDE methodology [39] and ISO 27001 risk management processes [40] can be used to describe and analyze threats. As a starting point for showing that pseudonymization protects data adequately, we propose to utilize methodologies developed in the area of privacy-preserving data outsourcing. In this field of computer science, it is studied how sensitive information can be protected when data is outsourced to untrusted entities, e.g. cloud providers [38, 41–45]. One protection mechanism that has been developed in this context, and which is very similar to pseudonymization, is data disassociation [41, 46]. With this method, data is distributed into different partitions and the data items as well the relationships between them are outsourced to different databases managed by different providers. In contrast to typical pseudonymization approaches, the properties of the partitioning and distribution of data are derived from formal threat models. In its most basic form, threats are expressed as so-called confidentiality constraints [45], which specify combinations of attributes that may not be accessible to an adversary in combination.

In this work, we focused on the implementation of pseudonymization into data collecting processes. In scenarios where re-identification is not a common procedure, e.g. when data is shared or used for secondary purposes, other aspects are likely to be of relevance and the trade-off between protection and complexity may be different.

## Additional file

Additional file 1: Literature Search and Selection Process. The file contains a detailed description of our literature search and selection process. (PDF 361 kb)

## Data Availability

The supplementary materials include a description of our literature search process.

## Abbreviations

HIPAA: Health Insurance Portability and Accountability Act
ISO: International Organization for Standardization
TLS: Transport Layer Security
UK: United Kingdom
