# Supplementary material for: Pseudonymization for research data collection: is the juice worth the squeeze?
Source: BMC Med Inform Decis Mak. 2019 Sep 4;19:178. doi: 10.1186/s12911-019-0905-x (PMC6727563; doi:10.1186/s12911-019-0905-x)
Supplement: Supplementary file 1 — Literature Search and Selection Process. The file contains a detailed description of our literature search and selection process. (PDF 361 kb) [file 12911_2019_905_MOESM1_ESM.pdf]

SUPPLEMENTARY FILE A: LITERATURE SEARCH AND SELECTION PROCESS

We employed a three-step process to select articles that can be used to get an impression of how aspects related to data pseudonymization are described in the literature. Our aim was to select articles that focus on concrete systems and which emphasize data privacy aspects.

We first performed searches on *PubMed*, the *ACM Digital Library*, *IEEE Xplore, Google Scholar* and *Springer*. We searched for articles which matched any reasonable combination of terms from three different groups in their title, abstract or in the keywords. Each group of terms described one specific aspect of medical data pseudonymization: (1) “medical”, “biomedical”, “clinical”, “health”, “genomic”, “genetic”, (2) “data”, “information”, “management”, “collection”, “storage”, (3) “privacy“, “protection”, “pseudonymization”, “pseudonyms“, “privacy“, “encryption”, “coding”, “reversible anonymization”, “pseudo-anonymization”, “key coding”, “honest broker” and “trusted third party”. We focused on articles published before August 2016.

In the second step, we then narrowed the set of articles down using eligibility criteria (ECs). To this end, the title, abstract and keywords of each article matching the search terms were analyzed individually by each of the three authors to determine whether they focus on the collection of pseudonymized biomedical data and/or biospecimens (EC-1). As our article focuses on the use of pseudonymization during research data collection, we did not consider articles focusing on the secondary use of health data. In the latter scenario, the degree of automatization is typically much higher (e.g. there is not necessarily a physician or researcher involved, which verifies the identity of the patient or proband) and privacy-preserving record linkage techniques can often be used that do not require a re-identification process (cf. the process implemented by the Synthetic Derivative at Vanderbilt UMC [19]). We further focused on articles published in English (EC-2). We also checked the search terms and ECs against all articles which were referenced by an article resulting from our primary search. This resulted in a collection of 28 articles.

In the third step, each article was read and analyzed by the three authors independently. We considered two additional criteria of eligibility. First, articles needed to describe concrete system instances, not only conceptual fragments (EC-3). This lead to the exclusion of the following articles: Refs. 1 - 13. Next, we required articles to list specific measures which have been implemented to protect the system from attacks (EC-4). This lead to the exclusion of the following three articles: Refs. 14 - 16. Finally, we obtained a selection of 12 articles, which were eligible for in-depth analysis. Overall, the following articles have been excluded from our analysis:

1. Churches T (2003) A proposed architecture and method of operation for improving the protection of privacy and confidentiality in disease registers. BMC Medical Research Methodology 3(1). doi:10.1186/1471-2288-3-1

2. De Moor GJE, Claerhout B, De Meyer F (2003) Privacy enhancing techniques: the key to secure communication and management of clinical and genomic data. Methods Inf Med 42(2):148–53. doi:10.1267/METH03020148

3. Claerhout B, De Moor GJE, De Meyer F (2002) Secure communication and management of clinical and genomic data: the use of pseudonymisation as privacy enhancing technique. Stud Health Technol Inform 95:170–5. doi:10.3233/978-1-60750-939-4-170

4. Riedl B, Grascher V, Neubauer T (2007) Applying a threshold scheme to the pseudonymization of health data. In Dependable Computing. PRDC 2007, pp 397-400. doi:10.1109/PRDC.2007.24

5. Winter A, Funkat G, Haeber A, Mauz-Koerholz C, Pommerening K, Smers S, et al (2007) Integrated information systems for translational medicine. Methods Inf Med 46:601–7. doi:10.1160/ME9063

6. Pommerening K, Sax U, Müller T et al (2008) Integrating eHealth and medical research: The TMF data protection scheme. In: Blobel B, Pharow P, Zvarova J, Lopez D (ed) eHealth: Combining health telematics, telemedicine, biomedical engineering and bioinformatics to the edge. Akademische Verlagsgesellschaft Aka GmbH. Berlin, pp 5–10

7. Neubauer T, Riedl B (2008) Improving patients privacy with pseudonymization. Stud Health Technol Inform 136:691–696

8. Slamanig D, Stingl C (2008) Privacy aspects of eHealth. ARES 2008 - 3rd Int Conf Availability, Secur Reliab, Proc IEEE 2008, pp 1226–1233. doi:10.1109/ARES.2008.115

9. Riedl B, Grascher V, Neubauer T (2008) A secure e-health architecture based on the appliance of pseudonymization. J Software 3(2):23–32. doi:10.4304/jsw.3.2.23-32

10. Veeningen M, De Weger B, Zannone N (2013) Formal modelling of (de) pseudonymisation: A case study in health care privacy. Secur Trust Manag, pp 145-160. doi:10.1007/978-3-642-38004-4_10

11. Bialke M, Penndorf P, Wegner T, Bahls T, Havemann C, Piegsa J, et al (2015) A workflow-driven approach to integrate generic software modules in a trusted third party. J Transl Med 13:176. doi:10.1186/s12967-015-0545-6

12. Arsenault S, Gaudet D (1997) Does a computer-based system help researchers in medical genetics to perform familial studies without manipulating nominal data? Proc AMIA Annu Fall Symp, American Medical Informatics Association, p 906

13. Jabeen F, Hamid Z, Wadood A, Ghouzali S, Khan A, Nawaz S, et al (2017). Enhanced Architecture for Privacy Preserving Data Integration in a Medical Research Environment. IEEE Access.

14. Noumeir R, Lemay A, Lina JM (2007) Pseudonymization of radiology data for research purposes. J Digit Imaging 20(3):284–295. doi:10.1007/s10278-006-1051-4

15. Muscholl M, Lablans M, Wagner TO, Ückert F (2014) OSSE: open source registry software solution. Orphanet J Rare Dis. 9 Suppl 1. doi:10.1186/1750-1172-9-S1-O9

16. Gulcher JR, Kristjánsson K, Gudbjartsson H, Stefánsson K (2000) Protection of privacy by third-party encryption in genetic research in Iceland. Eur J Hum Genet 8:739–742
